# Evaluation of the efficacy of transfer energy capacitive and resistive therapy in patients with knee osteoarthritis

**DOI:** 10.55730/1300-0144.5913

**Published:** 2024-10-07

**Authors:** Özge TEZEN, Emine Esra BİLİR, Öznur UZUN, Duygu YANIKTAŞ, Başak ŞENTÜRK, Evren YAŞAR

**Affiliations:** Department of Physical Medicine and Rehabilitation, Ankara Bilkent City Hospital, Ankara, Turkiye

**Keywords:** Knee osteoarthritis, transfer energy capacitive and resistive therapy, isometric quadriceps strength

## Abstract

**Background/aim:**

This study aimed to compare the therapeutic efficacy of conventional physical therapy (CPT) methods for knee osteoarthritis (OA) and transfer energy capacitive and resistive (TECAR) therapy, a relatively new and increasingly used treatment modality, based on patient clinical outcomes assessments.

**Materials and methods:**

Two groups of 54 patients, aged 40 to 75, were randomly assigned. CPT was given to both groups. In addition to CPT, Group 2 underwent TECAR therapy for six sessions, three times a week for two weeks. The Western Ontario and McMaster Universities Arthritis Index (WOMAC) was used to measure the disability and pain levels of each patient before and at the end of treatment and at 1 month and 3 months. Additionally, goniometric measurements of each patient’s knee joint range of motion and isometric quadriceps muscle strength were taken.

**Results:**

Significant improvements were noted in the VAS, WOMAC, and isometric quadriceps strength ratings in both groups between the pre- and posttreatment follow-ups. However, there was no discernible difference between the groups.

**Conclusion:**

For the conservative treatment of OA in the knee, TECAR therapy may be a helpful therapeutic approach.

## 1. Introduction

Osteoarthritis (OA) is a degenerative joint disease mainly affecting older people. It is defined by the erosion of articular cartilage, bone edges (osteophytes) enlargement, and biochemical and morphological alterations in the synovium and joint capsule [1]. OA most commonly occurs in weight-bearing joints, and the knee joint is the most commonly affected peripheral joint in OA. For those suffering from OA, pain, joint stiffness, and loss of function resulting from degeneration of the articular cartilage can impair quality of life and lead to significant morbidity [2].

The symptoms of knee OA, which is a chronic condition, include joint pain and functional impairment. It affects adults over 50 far more frequently. Pharmacological and nonpharmacological treatment is the most common treatment approach for mild to moderate knee OA. Patients should consider surgical joint replacement if they have considerable functional loss and ongoing pain. The majority of international guidelines advise individuals with knee OA to participate in a regular rehabilitation program to manage their pain and disability [3].

While there is currently no definitive medical cure for OA, there are ways to improve patients’ quality of life by reducing pain, increasing mobility, and reducing disability. For knee OA, nonpharmacological therapy options include weight loss, regular exercise, education, and the use of assistive equipment, including canes, insoles, and knee braces; in addition, physical therapy modalities, such as low-dose laser therapy, transcutaneous electrical nerve stimulation (TENS), hot packs (HP), short wave diathermy (SWD), ultrasound (US), and transfer energy capacitive and resistive (TECAR) therapy, are several new treatment applications that have become increasingly widespread in recent years [3,4].

TECAR therapy is a noninvasive method used in different musculoskeletal system pathologies, such as lower back pain, knee pain, Achilles tendon pathologies, and rotator cuff tendon pathologies. Its effectiveness in improving sports injuries and athlete performance, pelvic floor problems, lymphedema, and visceral pathologies like chronic constipation has been investigated, and it has been reported that it provides improvement in various parameters [5,6].

TECAR therapy is an endogenous diathermy treatment that heats the treated tissues using radiofrequency energy with a long wavelength (0.5 MHz). Capacitive (CAP) and resistive (RES) are the two treatment modes offered by the TECAR device. Depending on the treated tissue’s resistance, the two treatment techniques cause distinct tissue reactions. Muscle, cartilage, adipose tissue, and the lymphatic system are examples of water-rich, low-impedance soft tissues that are selectively affected by the energetic transmission of CAP when a ceramic layer acts as a dielectric medium and covers the active electrode. Only heat is produced in these superficial tissue layers. If the insulating layer on the active electrode is absent, radiofrequency energy from the RES travels directly through the body to the inactive electrode, heating the lower-lying, more resistant layers of tissue (muscle fascia, capsules, tendons, bone) deeper in the body [3,4].

To our knowledge, there are a limited number of studies investigating the effects of TECAR therapy on knee OA.

The current study aimed to compare the effectiveness of conventional physical therapy (CPT) applications and the modern application of TECAR therapy, which has gained popularity recently, in treating knee OA. The efficiency of the procedure was determined by analyzing the clinical findings of two patient groups.

## 2. Material and methods

### 2.1. Study design and participants

Between June 2023 and August 2023, Ankara City Hospital Physical Therapy and Rehabilitation Hospital’s outpatient clinic conducted this prospective, randomized clinical investigation. The study comprised 54 individuals aged 40 to 75 who reported knee discomfort and who, based on the criteria set forth by the American College of Rheumatology, were diagnosed with primary knee OA. Inclusion criteria were as follows: for nonresponsive knee pain, 3 months of conservative care, a radiographic evaluation grade between 2–4 on the Kellgren–Lawrence scale, and consent to be included in the trial. The exclusion criteria were the following: history of intraarticular injection therapy and physical therapy within the last 3 months; history of trauma or surgery to the injured knee; neurological or inflammatory condition affecting the lower limbs; severe peripheral vascular disease or active vasculitis; breastfeeding or pregnancy; restless legs syndrome diagnosis; local sensory impairment; neoplasia; acute infections, either systemic or local; presence of a pacemaker; psychiatric disorder; and cognitive impairment. The guiding principles of the Declaration of Helsinki were adhered to throughout the study’s implementation. The Ankara City Hospital Ethics Committee approved the study protocol (No. E2-23-3792).

Demographic data (age, sex, occupation), complaints, medical history, physical examination findings, and radiological examination results were prepared and recorded on a special form. The patients’ medical histories were assessed for diseases that may lead to secondary gonarthrosis, such as trauma, bone diseases, and inflammatory diseases.

**Figure 1:**
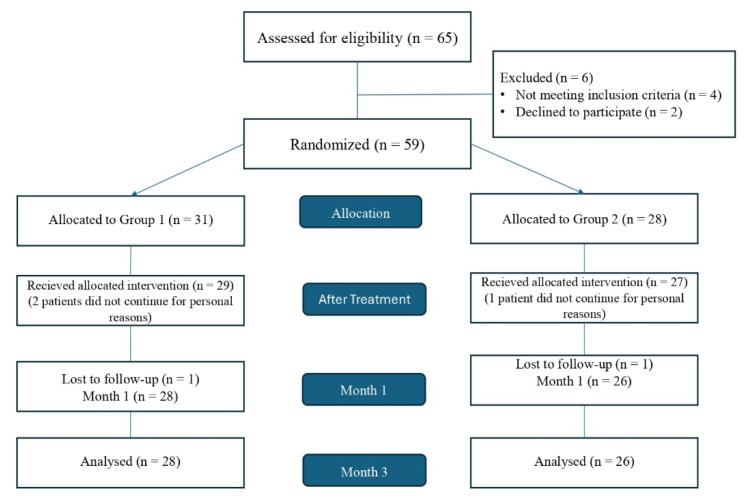
Flow chart of the study process.

Randomization was performed using the closed envelope method in the first assessment of the patients before treatment. The flow chart in Figure 1 shows the participant selection process.

### 2.2. Treatment protocols

For two weeks, five times a week, both groups received ten sessions of TENS and HP. Applying a heating pad to provide superficial warmth for 20 min, HP was applied to the painful knee area. A Chattanooga-brand device was used to apply TENS to the bothersome knee area for 20 min at a frequency of 80 Hz. At the same time, both groups received knee isometric and Theraband exercise therapy with the help of a physiotherapist. TECAR therapy was additionally given to Group 2 patients by a physiotherapist trained in this field for a total of six sessions, which is three times a week for two weeks, using the BTL-6000 TR-THERAPY device at a frequency of 0.5 MHz in 5 min CAP + 10min RES + 5 min CAP mode. The return (inactive) electrode was placed on the opposite calf, between the head of the fibula and the lateral malleolus. No side effects, such as skin redness or burning, were observed in the patients during treatment.

### 2.3. Clinical and radiological assessments

Goniometric measurements were made of the range of motion (ROM) of each patient’s knee joint. The Western Ontario and McMaster Universities Arthritis Index (WOMAC) scale was used to measure disability, the Visual Analog Scale (VAS) was used to score pain, and the Diers myoline isometric muscle strength measurement system was used to measure isometric quadriceps muscle strength. One researcher assessed each patient before therapy, after treatment, and at 1 and 3 months; the researcher was blinded to the treatment groups.

The intensity of pain was measured utilizing a 10-centimeter VAS. Participants indicated their average pain level throughout the preceding 24 h (including periods of rest, motion, and during the night) by marking a point on the scale, which ranged from 0 (no pain) to 10 (worst pain imaginable). The VAS is a widely accepted and dependable tool for assessing pain [7,8]. Typically, a reduction of approximately 1.9–2.0 points on the VAS (equating to a 19–20% decrease) is deemed clinically significant [9].

The WOMAC global score was employed to assess the functional aspect of life quality. A self-reported questionnaire—the WOMAC—is used to gauge how well individuals with hip or knee OA are feeling in terms of pain, stiffness, and physical function. Each of the three subscales in this 24-item index measures pain, stiffness, and physical function and is scored on a five-point Likert scale from “none” to “extreme.” Significant improvement is defined as a 16% decrease from the baseline WOMAC global score [10,11].

The ROM of the knee joint was evaluated with participants lying supine, ensuring that this position was within their pain tolerance. Flexion and extension were measured using a universal goniometer, with reference to bony landmarks, including the greater trochanter, lateral femoral condyle, fibular head, and lateral malleolus. Universal goniometry is widely recognized as a dependable and established method for promptly assessing joint ROM [12].

The Diers Myoline Isometric Muscle Strength Measurement System (Schlangenbad, Germany, 2019) was used to obtain isometric quadriceps muscle strength measurements. Patients were seated in a chair with their backs supported and arms placed comfortably on the armrests. Their knees were extended over the edge of the seat and secured with straps around their shoulders, waist, and ankles to provide stability during testing. After positioning the patients, the test procedure was explained in detail using a visual feedback screen. The patients were then asked to perform maximum knee extension and hold the muscle isometrically for 10 s. The test was repeated twice, and the third test was used for measuring and recording the results.

### 2.4. Statistical analysis

SPSS version 25.0 (IBM Corp., Armonk, NY, USA) was used to analyze the data. The Shapiro–Wilk normality test was employed to determine whether the numerical data distribution was normal. Standard deviation was used to represent continuous variables with a normal distribution, median and interquartile range (IQR: 25th–75th percentile) was used to represent nonnormally distributed data, and frequency and percentage were used to represent qualitative data. The chi-square test was employed to compare the categorical data, and Fischer’s exact’s continuity correction test was used based on the frequency of expected counts. Using an independent samples t-test or a Mann–Whitney U test, numerical variables with parametric or nonparametric distributions between the groups were compared. The Wilcoxon signed-rank test was utilized to compare variables with nonnormal distribution that were measured repeatedly. The group influence on changes in WOMAC and isometric quadriceps muscle strength scores over time was examined using a two-way ANOVA test. The margin of error allowed was 5%, and the confidence interval was 95%; p < 0.05 was the threshold for statistical significance. The sample size calculation was performed considering the VAS pain score as the primary outcome measure and adopting the mean and standard deviation from a previously published study in patients with knee OA. Accordingly, using G*Power software (v. 3.1), the minimum sample size was calculated as 19 patients per group (38 patients in total) for a power of 0.80 and an effect size of 0.83 with an alpha error of 0.05 [13].

## 3. Results

In total, 30 female and 24 male patients made up the sample for this study. Patients were allocated to two groups: Group 1 = 28; Group 2 = 26. The demographic features of the groups are presented in Table 1. The groups were similar in terms of age, sex, and body max index (for all features: p > 0.05). The groups were not significantly different in terms of systemic disease presence, duration of pain, and Kellgren–Lawrence stage (for all features: p > 0.05). The clinical characteristics of the groups are given in Table 2.

The VAS, WOMAC, and isometric quadriceps strength scores of the groups are shown in Table 3. Significant improvements were observed in both groups in posttreatment follow-up compared to pretreatment. However, there was no significant difference between the groups. The VAS night pain score in Group 2 was lower at 1 month compared to Group 1 (p = 0.034).

The p-values of the VAS scores over time compared to before the treatment period are shown in Table 4. For Group 1, the VAS rest, motion, and night scores were significantly lower at the end of treatment and at the 1st and 3rd months than during pretreatment (p < 0.05). For Group 2, the VAS rest and motion scores were significantly lower at the end of the treatment period and at the 1st and 3rd months than before treatment (p < 0.05). However, the VAS night scores were significantly lower in Group 2 at the end of the treatment period and at the 1st month compared to before treatment (p < 0.05); however, no significant difference was found between groups in the 3rd-month and pretreatment scores (p = 0.165).

**Figure 2:**
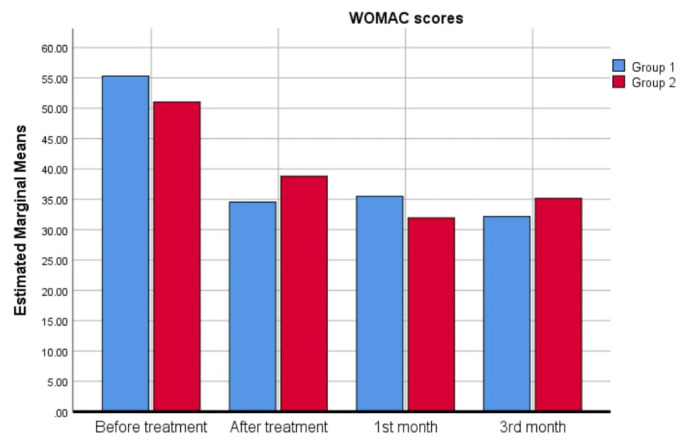
WOMAC scores of the groups over time.

When the time-dependent change in WOMAC scores was analyzed, a significant decrease was observed in both groups at the end of treatment compared to before the treatment period (p < 0.001); therefore, there was no group effect on time-dependent changes (p = 0.973). This reduction of WOMAC scores continued until the 3rd month after treatment. No significant difference existed in the WOMAC scores between the end of treatment and the 1st month (p = 0.153) and between the 1st and 3rd months (p = 0.977). The changes in WOMAC scores over time are illustrated in Figure 2.

**Figure 3:**
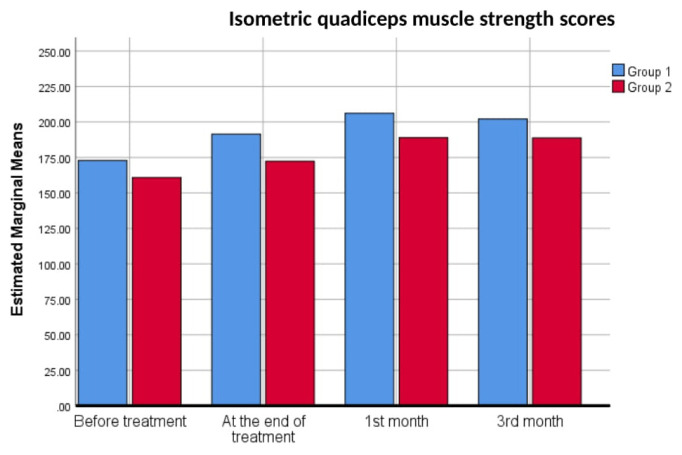
Isometric quadriceps muscle strength scores of the groups over time.

In terms of pre- and posttreatment measurements, there was a significant change in isometric quadriceps muscle strength in both groups (p < 0.001). Therefore, no group effect was found on the increase in isometric muscle strength scores (p = 0.525). The 1st-month scores were significantly higher than the posttreatment scores (p = 0.043). There was no significant difference between pretreatment and end of treatment (p = 0.093) and between 1st- and 3rd-month scores (p = 0.486). The changes in isometric quadriceps muscle strength scores over time are shown in Figure 3.

## 4. Discussion

Although OA of the knee is a prevalent degenerative joint disease, a consensus regarding treatment does not exist [1,2]. However, there is evidence that some diathermy treatments (e.g., US, SWD) can be beneficial. In recent years, TECAR therapy has become a prominent method to alleviate knee OA symptoms and improve patients’ functionality [3]. The study’s objective was to assess and compare how TECAR therapy for treating knee OA affected knee pain, muscular strength, disability, and range of motion.

The administration of high-frequency electromagnetic waves is the mechanism of action of TECAR therapy; it decreases muscular contractions and spasms, increases blood flow, and increases muscle oxygenation through hemoglobin activation.

TECAR therapy can assist the body’s natural healing processes, potentially leading to tissue repair and pain reduction [14,15]. Pain due to knee OA is a well-known symptom of underlying degenerative and inflammatory processes. The current literature suggests that TECAR therapy may also be beneficial for knee OA due to its antiedematous and antiinflammatory effects, increased endorphin release, and enhanced cellular metabolism [16].

Several studies in the literature have indicated that TECAR treatment is effective [17–19]. In their meta-analysis, Vahdatpour et al. evaluated the effects of TECAR therapy on pain intensity in patients with musculoskeletal disorders. The authors reported a significant decrease in the patients’ VAS scores and an improvement in their quality of life [20]. Beltrame et al. also established that TECAR therapy for musculoskeletal disorders improved strength and function and reduced pain intensity [21]. Similar to the above two studies, the present study also found a decrease in pain scores, an improvement in muscle strength, and a reduction in disability in patients.

Ribeiro et al. suggested that TECAR therapy may be a good complementary treatment, either incorporated into a traditional rehabilitation program or applied alone, as it has both short and long-term advantages. The most significant advantage of this therapy is its usability during the acute phase, allowing for earlier treatment initiation and significantly reducing the risk of complications associated with immobilization or limited mobility [5]. In the present study, the pain intensity of the patients was evaluated using the VAS score. In Group 1, the VAS (rest-movement-night) pain scores were found to be significant at all follow-up sessions compared to pretreatment; however, for Group 2, while the night pain VAS score was more significant in the short term, no significant difference was observed between the two groups in the long term. The superior short-term effect of TECAR therapy on knee OA is attributed to its ability to directly target and heat these tissues using radio frequency currents—in contrast to superficial thermotherapy, which attempts to penetrate deep muscles. This goal-oriented approach can be explained by its endogenous therapy nature, targeting the underlying layers rather than the epidermis, thereby positively influencing blood flow, accelerating the elimination of waste products (catabolites), and dilating peripheral vessels, all of which contribute to its antiinflammatory and antiedematous effects [4,10,22].

Cocetta et al. observed significant improvements in the VAS and WOMAC scores in the TECAR group in knee OA, while no significant change was observed in the sham group [3]. Both groups in the current study received CPT and did exercise, but TECAR therapy was additionally given to Group 2. Consistent with the literature, this study demonstrated significant improvement in pain, stiffness, and physical function in both groups. However, we attribute the lack of TECAR therapy’s superiority over CPT in all outcomes—except for the early night VAS score—to the fact that the other group also received CPT.

In Kumaran et al.’s study, which investigated the effect of capacitive resistive monopolar radiofrequency on knee OA patients’ pain and function, the decrease in VAS and WOMAC scores was more significant than in the sham and control groups (exercise and education only) and continued at the 3-month follow-up. While the results of the ROM of the knee joint were significant only in the active group, the two other groups showed no discernible changes [9]. In the present study, both groups received CPT, and we found significant improvements in the VAS, WOMAC, and isometric muscle strength scores in both groups. While there was no statistically significant difference between the groups in the long term, only short-term VAS scores were better in the TECAR group compared to CPT. Since the patients had no significant limitations in their pretreatment knee ROM measurements, there was no significant difference in the follow-up measurements after treatment.

## 5. Limitations

The study has a few limitations. First, there was no sham group. This made it difficult to definitively conclude that the observed improvements were actually due to the applied treatment. Second, the small sample size is a limitation. Third, the sample group in the study was not followed up in the long term. The lack of evaluation with US or MRI before and after treatment, which would have provided more objective results, is another limitation.

## 6. Conclusion

This investigation was spurred by the scarcity of publications in the literature comparing TECAR therapy’s effectiveness in the management of knee OA. The results showed that TECAR is a useful treatment method in reducing pain and impairment and enhancing patients’ quality of life due to knee OA, similar to other conventional treatment methods. Further research is necessary to standardize therapeutic protocols for this patient group. TECAR therapy can be used alone or in combination with exercise and other physical therapy modalities. To determine the effectiveness and safety of this treatment, more investigation is necessary.

## Figures and Tables

**Table 1:** Demographic features of the groups.

	Group 1 (n = 28)	Group 2 (n = 26)	p

Age (mean ± SD)	62.36 ± 11.58	60.54 ± 8.16	0.511

Sex (n/%)			
Female	16 (57.1)	14 (53.8)	1.000
Male	12 (42.9)	12 (46.2)	

Marital status (n/%)			
Married	21 (75.0)	24 (92.3)	0.088
Single	2 (7.1)	1 (3.8)	
Widow	5 (17.9)	1 (3.8)	

Occupation (n/%)			
Officer	4 (14.3)	7 (26.9)	
Standing work	1 (3.6)	2 (7.7)	0.090
Housewife	2 (7.1)	4 (15.4)	
Retired	21 (75.0)	13 (50.0)	

Educational status (n/%)			
Primary school	7 (25.0)	6 (23.1)	
Secondary school	2 (7.1)	2 (7.7)	0.111
High school	7 (25.0)	1 (3.8)	
University	12 (42.9)	17 (65.4)	

BMI (mean ± SD)	28.53 ± 2.95	29.90 ± 5.24	0.246

BMI: Body Mass Index.

**Table 2:** Clinical characteristics of the groups.

	Group 1 (n = 28)	Group 2 (n = 26)	p

Systemic disease (n/%)	13 (46.4)	18 (69.2)	0.107

Previous analgesic use for pain relief (n/%)			
None	6 (21.4)	6 (23.1)	
NSAIDs	8 (28.6)	5 (19.2)	
Topical treatments	3 (10.7)	6 (23.1)	
HA preparate	3 (10.7)	2 (7.7)	
NSAIDs and topical treatments	7 (25.0)	3 (11.5)	
NSAIDs and HA preparate	-	1 (3.8)	
NSAIDs and topical treatments and HA	1 (3.6)	3 (11.5)	

Duration of pain	15 .0 (21.0)	24.0 (27.0)	0.361

Factor aggravating pain (n/%)			
Walking	12 (42.9)	10 (38.5)	
Walking upstairs	7 (25.0)	5 (19.2)	
Squatting	9 (32.1)	11 (42.1)	

Previous PMR program (n/%)	2 (7.1)	3 (11.5)	

Kellgren–Lawrence stage (n/%)			
Stage 2	9 (32.1)	12 (46.2)	0.543
Stage 3	17 (60.7)	13 (50.0)	
Stage 4	2 (7.1)	1 (3.8)	

NSAID: Nonsteroidal antiinflammatory drug, HA: Hyaluronic acid, PMR: Physical medicine and rehabilitation.

**Table 3:** Visual analog scale, Western Ontario and McMaster Universities Osteoarthritis Index, and isometric quadriceps strength scores of the groups.

		Group 1	Group 2	p
Before treatment	VAS-rest	3.0 (4.8)	3.5 (4.0)	0.413
	VAS-motion	8.0 (4.5)	7.0 (4.5)	0.476
	VAS-night	5.5 (5.5)	3.0 (3.3)	0.062
	WOMAC	55.32 ± 1.92	51.04 ± 21.25	0.426
	Isometric quadriceps muscle strength	172.82 ± 90.59	160.85 ± 63.59	0.579
The end of the treatment	VAS-rest	1.0 (3.8)	2.0 (4.0)	0.888
	VAS-motion	4.0 (4.8)	4.0 (5.0)	0.238
	VAS-night	2.0 (5.0)	2.0 (4.3)	0.652
	WOMAC	34.54 ± 16.66	38.79 ± 21.22	0.416
	Isometric quadriceps muscle strength	191.41 ± 111.84	172.31 ± 82.83	0.482
At 1^st^ month	VAS-rest	1.0 (2.5)	1.0 (3.0)	0.284
	VAS-motion	4.0 (3.0)	3.0 (3.3)	0.186
	VAS-night	2.0 (2.0)	1.0 (1.5)	**0.034**
	WOMAC	35.49 ± 18.00	31.94 ± 18.15	0.474
	Isometric quadriceps muscle strength	206.18 ± 120.19	188.92 ± 74.00	0.532
At 3^rd^ month	VAS-rest	1.0 (2.0)	1.5 (2.3)	0.436
	VAS-motion	4.0 (3.5)	4.0 (3.5)	0.820
	VAS-night	3.0 (3.8)	2.0 (2.3)	0.916
	WOMAC	32.15 ± 18.31	35.16 ± 16.78	0.541
	Isometric quadriceps muscle strength	202.11 ± 113.36	188.73 ± 70.51	0.608

VAS: Visual analog scale, WOMAC: Western Ontario and McMaster Universities Osteoarthritis Index.

**Table 4:** Comparison of visual analog scale scores at different times compared to before the treatment period.

	The end of treatment and before treatment	1^st^ month and before treatment	3^rd^ month and before treatment
**Group 1**	p		
VAS-rest	**<0.01**	**<0.01**	**<0.001**
VAS-motion	**<0.01**	**<0.001**	**<0.001**
VAS-night	**<0.01**	**<0.001**	**<0.001**
**Group 2**			
VAS-rest	**<0.01**	**<0.001**	**<0.01**
VAS-motion	**<0.01**	**<0.001**	**<0.01**
VAS-night	**<0.01**	**<0.01**	0.165

VAS: Visual analog scale.
